# Ascites Bacterial Burden and Immune Cell Profile Are Associated with Poor Clinical Outcomes in the Absence of Overt Infection

**DOI:** 10.1371/journal.pone.0120642

**Published:** 2015-03-17

**Authors:** Kevin J. Fagan, Geraint B. Rogers, Michelle Melino, Dionne M. Arthur, Mary-Ellen Costello, Mark Morrison, Elizabeth E. Powell, Katharine M. Irvine

**Affiliations:** 1 Centre for Liver Disease Research, School of Medicine, The University of Queensland, Translational Research Institute, Brisbane, Australia; 2 Department of Gastroenterology and Hepatology, Princess Alexandra Hospital, Brisbane, Australia; 3 SAHMRI Infection and Immunity Theme, School of Medicine, Flinders University, Adelaide, Australia; 4 The University of Queensland Diamantina Institute, Translational Research Institute, Brisbane, Australia; University of Sydney, AUSTRALIA

## Abstract

Bacterial infections, most commonly spontaneous bacterial peritonitis in patients with ascites, occur in one third of admitted patients with cirrhosis, and account for a 4-fold increase in mortality. Bacteria are isolated from less than 40% of ascites infections by culture, necessitating empirical antibiotic treatment, but culture-independent studies suggest bacteria are commonly present, even in the absence of overt infection. Widespread detection of low levels of bacteria in ascites, in the absence of peritonitis, suggests immune impairment may contribute to higher susceptibility to infection in cirrhotic patients. However, little is known about the role of ascites leukocyte composition and function in this context. We determined ascites bacterial composition by quantitative PCR and 16S rRNA gene sequencing in 25 patients with culture-negative, non-neutrocytic ascites, and compared microbiological data with ascites and peripheral blood leukocyte composition and phenotype. Bacterial DNA was detected in ascitic fluid from 23 of 25 patients, with significant positive correlations between bacterial DNA levels and poor 6-month clinical outcomes (death, readmission). Ascites leukocyte composition was variable, but dominated by macrophages or T lymphocytes, with lower numbers of B lymphocytes and natural killer cells. Consistent with the hypothesis that impaired innate immunity contributes to susceptibility to infection, high bacterial DNA burden was associated with reduced major histocompatibility complex class II expression on ascites (but not peripheral blood) monocytes/macrophages. These data indicate an association between the presence of ascites bacterial DNA and early death and readmission in patients with decompensated cirrhosis. They further suggest that impairment of innate immunity contributes to increased bacterial translocation, risk of peritonitis, or both.

## Introduction

Infections are responsible for much of the morbidity, mortality and resource utilization in patients with decompensated cirrhosis[1,2]. Bacterial infections, most commonly spontaneous bacterial peritonitis (SBP) in patients with ascites, occur in one-third of admitted patients with cirrhosis, and account for a 4-fold increase in mortality[3], but absence of clinical signs of infection is frequent and may delay diagnosis and treatment. Less than 40% of ascites infections are culturable, requiring initiation of empirical antibiotic treatment. The mortality rate after infection in cirrhosis remains high (28.6% at 1 month, 63% at 1 year) and has not changed substantially over recent decades[3]. However, culture-independent studies suggest bacteria are commonly present, even in the absence of overt infection. A combination of 16S rRNA gene sequencing and quantitative PCR was recently used to show that ascitic fluid from cirrhotic patients comprises a continuum from low-level bacterial colonization in the absence of a neutrophil response, through to clinically significant and severe SBP[4]. Although substantial variation in the bacterial species detected was observed between patients, microbiota community membership and structure correlated with differences in ascitic fluid neutrophil count and patient Child-Turcotte-Pugh (CTP) class[4]. The widespread detection of low levels of bacteria in ascites in the absence of peritonitis suggests first, that bacterial translocation to the peritoneal cavity is a common process, and second, that the entry of bacteria into this site may not be sufficient to give rise to SBP. Here, host immune impairment may also contribute to the risk of SBP in some cirrhotic patients. However, little is currently known about the role of ascites leukocyte composition and function in this context.

Innate immune cells, especially monocytes/macrophages, represent the first line of defence against microbes. Various defects in peripheral monocytes have been described in chronic liver disease (CLD)[2], including in anti-bacterial effector functions, similar to the “immune paralysis” observed in sepsis. Monocyte deactivation in patients with decompensated cirrhosis directly influences outcomes, and is a tractable therapeutic target[2,5]. However, monocyte deactivation is likely to change over time, and differ between anatomical sites. Ascitic fluid provides a unique portal through which immune function can be assessed at the site of infection, but ascites leukocytes have been surprisingly little studied. Moreover, ascitic fluid has been reported to contain (unidentified) immune inhibitory factors[6]. The extent of immunoparalysis in ascites, and the relative contribution of cell intrinsic and cell extrinsic factors, is not known.

The first aim of this study was to quantify bacterial DNA in ascitic fluid, in order to determine whether bacterial burden is associated with clinical outcomes, including infection, survival or incidence of decompensation events (upper gastrointestinal bleeding, hepatic encephalopathy, hepatocellular cancer). The second aim was to characterise ascitic fluid and peripheral blood leukocytes, to determine the extent to which immune phenotype is site-specific, and its relationship to microbial burden, clinical parameters and outcomes.

## Experimental Procedures

### Patients and clinical data

Ascitic fluid and matched peripheral blood samples were obtained from 25 patients with decompensated cirrhosis undergoing paracentesis. Informed written consent was obtained from each patient and the protocol was approved by the Metro South Health and The University of Queensland Human Research Ethics Committees. Standard biochemical and serological assays, liver imaging and histological assessment of a liver biopsy (if performed) were used to confirm diagnosis of liver disease and cirrhosis. Liver disease severity was evaluated using the CTP classification[7], and Model for End-Stage Liver Disease (MELD)[8]. Patient medical records were reviewed to obtain demographic details, previously diagnosed liver disease and other medical conditions, medications and history of tobacco and alcohol use for each patient. Current alcohol use was defined as ‘significant’ if the patient consumed greater than the threshold of alcohol likely to cause liver injury, based on epidemiological data (140g/week for women, 210g/week for men)[9]. Heavy alcohol use was defined as ≥350g/week for women and ≥420g/week for men, for >6 months. Blood was drawn at the time of paracentesis for routine laboratory tests/blood cultures, or processed and stored at -20°C for analysis of C-reactive protein (Beckman DXC800), and procalcitonin (Mini Vidas). The serum-ascites albumin gradient (SAAG) was calculated by subtracting the albumin concentration of the ascitic fluid from the albumin concentration of a serum specimen obtained on the same day. SBP was defined as an ascitic fluid polymorphonuclear leukocyte count ≥250/mm^3^. Clinical outcome data for the 6 months after the paracentesis (survival, liver transplantation, readmission, incidence of SBP or decompensation events) were obtained from medical records.

### Cell isolation and DNA purification from ascites samples

14ml Ascites fluid was centrifuged at 5000x*g* for 10 minutes in sterile 15ml conical tubes. DNA was extracted from cell pellets using the QIAamp DNA Mini kit in accordance with the manufacturer’s instructions (Qiagen, Venlo, NL), resuspended in 100μl sterile water, and quantified using a Nanodrop spectrophotometer (Thermo Scientific, Waltham, MA, USA).

### Assessment of bacterial density

A real-time PCR assay that amplifies a 286 base pair region of the V4 region of the 16S rRNA gene was used to quantify bacterial DNA. PCR primers used were 517F (5′-GCCAGCAGCCGCGGTAA-3′) and 803R (5′- CTACCRGGGTATCTAATCC-3′)[10]. The total volume for each reaction was 20μl, containing 1x SYBR Select Master Mix (Applied Biosystems, Mulgrave, Australia), 1μl of 0.5μM forward and reverse primers and 50ng of template DNA. PCR was performed using an Mx3000P thermal cycler (Agilent Technologies, Santa Clara, USA) with the following cycling conditions: 50°C for 2 min, 95°C for 2 min, and 40 thermal cycles of 95°C for 15 sec; 57°C for 30 sec; 60°C for 30 sec. A final step of 95°C for 1 min and a dissociation curve protocol from 55° to 95° were performed. Phosphate buffered saline (PBS) that had been through the DNA extraction process was used as a negative control. PCR threshold cycles were used to calculate the amount of bacterial DNA in ascitic fluid (ng/μl) using a standard curve generated from purified *Escherichia coli* DNA and converted to an estimate of colony forming units (CFU)/ml.

### 16S rRNA amplicon sequencing

Sequencing of the V4 region of the 16S rRNA gene amplicon was carried out by MrDNA.com (Texas, USA) using primers 515F: 5′-GTGCCAGCMGCCGCGGTAA-3′ and 806R: 5′-GGACTACVSGGGTATCTAAT-3′ primers[10]. In brief, a single-step 30 cycle PCR using HotStarTaq Plus Master Mix Kit (Qiagen, Valencia, CA) was performed under the following conditions: 94°C for 5 minutes, followed by 28 cycles of: 94°C for 30 sec, 53°C for 40 sec, and 72°C for 1 min. Amplification was followed by a final elongation step at 72°C for 5 minutes. Following PCR, all amplicon products from different samples were mixed in equal concentrations and purified using Agencourt Ampure beads (Agencourt Bioscience Corporation, MA, USA). Sequencing was performed using the Roche 454 FLX titanium instruments and reagents according to manufacturer’s instructions[11]. Sequencing analysis was performed as previously described[12,13].

### Cell isolation and flow cytometric analysis

Cells were pelleted from ascites fluid at 500x*g* for 5 minutes, washed once with low glucose Dulbecco’s modified eagle medium (Invitrogen), and resuspended in freezing medium (10% DMSO (Sigma-Aldrich, Castle Hill, Australia), 50% fetal bovine serum (FBS, Invitrogen)), and stored in liquid nitrogen. Peripheral blood mononuclear cells (PBMC) were isolated using Ficoll density centrifugation, resuspended in freezing medium, and stored in liquid nitrogen. For flow cytometric analysis, 0.5-1x10^6^ PBMC or ascites cells were stained in 50μl PBS/2%FBS with a panel of antibodies comprising HLA-DR-FitC, CD56-PE, CD66B-PerCp.Cy5.5, CD3-AF700, CD19-APC, CD16-APC.H7, CD14-BV421 (leukocyte panel) or HLA-DR-FitC, CD11C-PE, CD163-PerCp.Cy5.5, CX3CR1-PE.Cy7, CCR2-APC, CD16-APC.H7, CD14-BV421 (monocyte panel) or single antibodies for compensation (Biolegend, San Diego, CA, USA or Becton Dickinson, Franklin Lakes, New Jersey, USA). Flow cytometry analysis was performed on a Gallios flow cytometer and data analysed using Kalluza (Beckman Coulter, Brea, CA, USA). Cell populations were gated based on unstained controls and single positive controls. Additionally, fluorescence-minus-one controls were used to verify background fluorescence levels during protocol development. Negative myeloid and lymphoid populations were gated separately as myeloid (SSC^Hi^) cells exhibited increased autofluorescence compared to lymphocytes. CD14^Hi^ cells formed an obvious, distinct population in all donors, facilitating their gating. A second, distinct SSC^Hi^ population was observed in the majority of donors, which apparently comprised CD14^Low^ and CD14^Negative^ cells, based on negative controls, but could not be clearly distinguished on the basis of CD14 expression.

### Statistical analysis

Statistical analysis of clinical and flow cytometry data was performed in Prism 6.04 (GraphPad, La Jolla, California, USA). Spearman’s correlation was used to compare continuous variables, and Mann Whitney U tests were used for group comparisons. A significance threshold of p<0.05 was used. Several statistical tools were used to assess whether relationships existed between bacterial community composition and clinical factors. Measures of bacterial community diversity (genus richness, Simpson index, and Shannon index) were assessed using PAlaeontological STatistics, version 3.01 (PAST), available from the University of Oslo (http://folk.uio.no/ohammer/past). Non-metric Multidimensional scaling (NMS) based on Bray-Curtis (BC) similarity measures, one-way ANOSIM tests, were used to assess whether bacterial community composition differed significantly between groups according to categorical variables (etiology, previous SBP, prophylactic antibiotic use, antibiotic treatment within preceding 14 days, and 6 month mortality) using PAST.

## Results

### Patient characteristics at paracentesis

Twenty five patients undergoing paracentesis for ascites secondary to chronic liver disease of various etiologies, principally alcohol and hepatitis C virus (HCV) infection, were recruited (Table 1). The median age of the cohort was 55.4 years, 76% were male and 96% Caucasian. Eighteen patients admitted previous heavy alcohol consumption, although only 7 reported significant alcohol consumption during the prior year, 3 within the 2 weeks before the current paracentesis. The median number of prior paracenteses was 4. Over the 6 month follow-up period 2 patients developed their first episode of SBP, 9 patients died (1 during admission for SBP, 1 from pneumonia) and 3 received a liver transplant. Seven patients died with decompensated cirrhosis, however the contribution of bacterial infections to their decompensation and death could not be accurately determined. There were 64 hospital admissions over the 6 month follow up period, in addition to 44 presentations to the day procedure unit for paracentesis.

**Table 1:** Patient demographic data and comorbidities at the time of ascitic fluid collection and outcomes during the 6 months follow up.

	Patient Cohort n = 25
**At Paracentesis**	
Age (years) median (IQR)	55.4
	(50.0–64.1)
Gender (n, % male)	19 (76)
Ethnicity (n, % Caucasian)	24 (96)
Etiology of cirrhosis (n, %)	
Alcohol	11 (44)
Hepatitis C virus	9 (36)
Other^c^	5 (20)
Previous SBP (n, %)	8 (32)
Previous evidence of gastroesophageal varices (n, %)	12 (48)
Previous evidence of hepatic encephalopathy (n, %)	7 (28)
Hepatocellular carcinoma (n, %)	3 (12)
Previous heavy^a^ alcohol consumption (n, %)	18 (72)
Significant^b^ alcohol consumption in last year (n, %)	7 (28)
Days since previous paracentesis, median (IQR)	147 (4–856)
Number of previous paracenteses, median (IQR)	4.0 (0–29)
**Outcomes (6 months)**	
Death (n, %)	9 (36)
Developed SBP (n, %)	2 (8)
Liver transplant (n, %)	3 (12)

^a^Previous heavy alcohol use was defined as ≥350g/week for women and ≥420g/week for men, for >6 months

^b^Significant alcohol was defined as >140g/week for women and >women and >210g/week for men

^b^Other: Non-alcoholic fatty liver disease (n = 1), cryptogenic cirrhosis (n = 1), primary biliary cirrhosis (n = 1), primary sclerosing cholangitis (n = 1), autoimmune hepatitis (n = 1).

### Bacterial DNA burden in ascitic fluid in the absence of overt infection is associated with poor clinical outcomes

**Fig 1:**
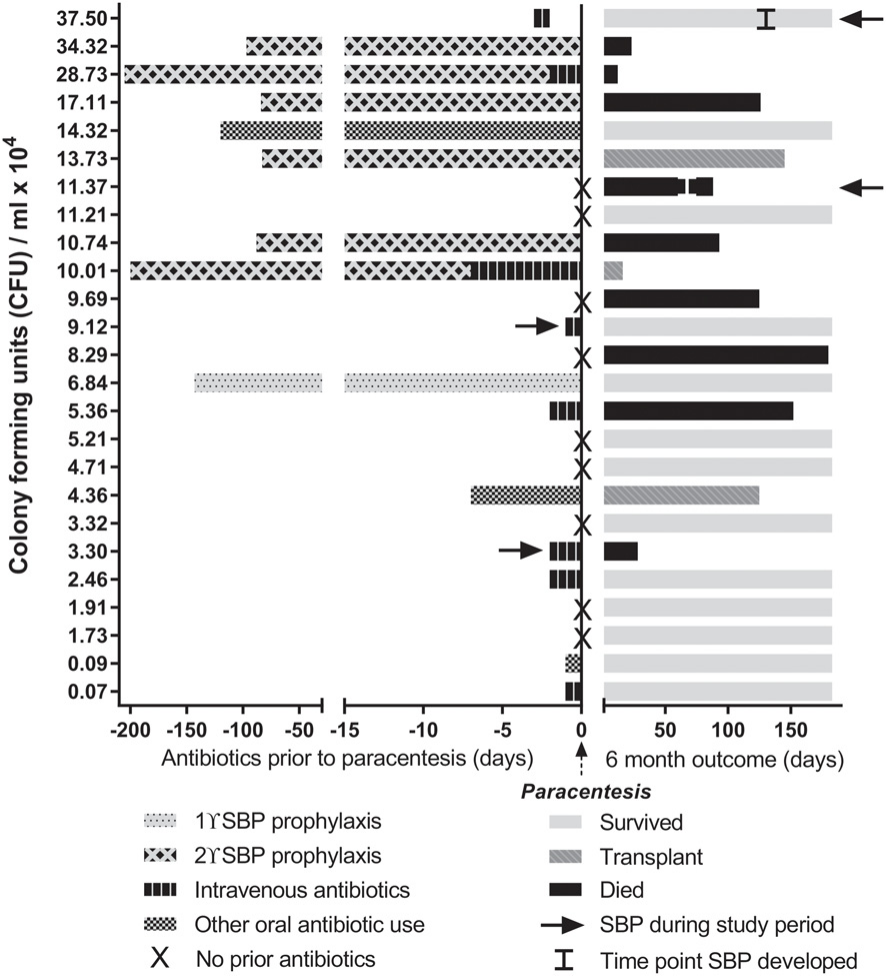
Ascites cohort antibiotic history and 6-month outcomes. Antibiotic treatment history prior to the study paracentesis and 6 month outcomes are depicted for each patient, in relation to their ascitic bacterial DNA burden (CFU/ml).

**Fig 2:**
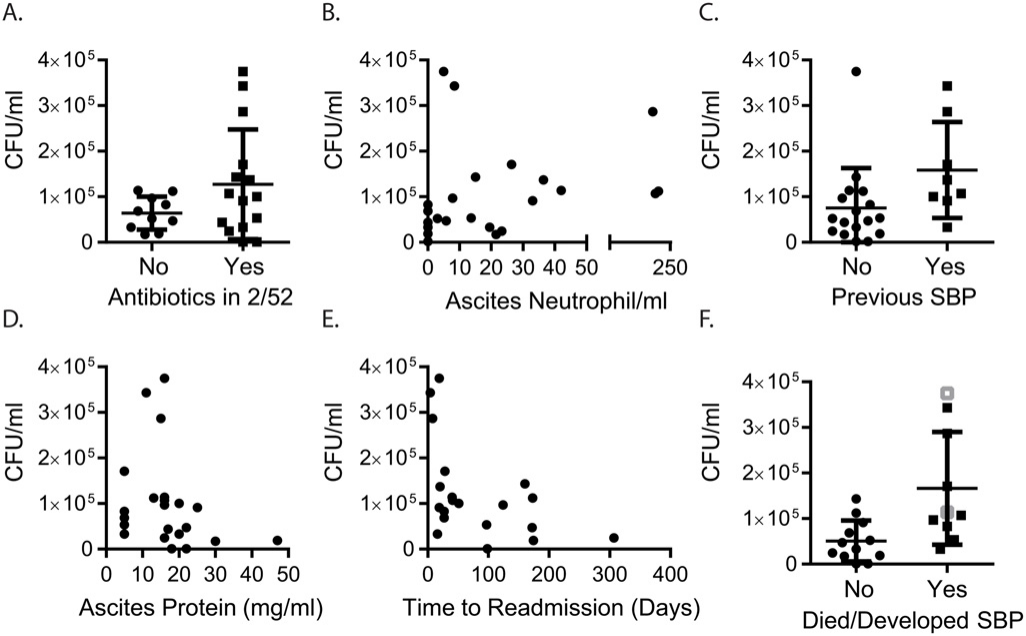
Bacterial DNA burden in ascitic fluid is associated with poor clinical outcomes. (A) Ascitic bacterial DNA burden in patients who had received antibiotics within the previous 2 weeks (2/52) (p = 0.28). (B) Correlation between bacterial DNA burden and the number of neutrophils/ml ascitic fluid (r_s_ = 0.5, p = 0.012). (C) Bacterial DNA burden in patients with a previous history of SBP (p = 0.027). Correlation between bacterial DNA burden and (D) ascites total protein content (r_s_ = -0.42, p = 0.045) and (E) time to hospital readmission (r_s_ = -0.50, p = 0.024). (F) Bacterial DNA burden in patients who survived and those who died (black squares) or developed SBP (grey squares, # developed SBP and died, p = 0.006).

Bacterial 16S rRNA DNA (hereafter ‘16S’) was detectable in 23 of 25 patients, at levels equivalent to 0.09–1.9 ng/μl *E*.*coli* DNA, which would equate to approximately 10^3^–10^5^ *E*.*coli* CFU/ml (CFU equivalents/ml, hereafter ‘CFU/ml’). Clinical histories of this cross-sectional cohort were diverse; many patients had prior events expected to influence gut and/or ascites microbiota, including previous paracenteses, SBP, and antibiotic treatment (summarised in Fig. 1, relative to ascites bacterial DNA levels). Two patients were diagnosed with SBP (ascites neutrophil count ≥250/mm^3^) 2 days prior to paracentesis, but none of the patients had an ascites neutrophil count ≥250/mm^3^, positive bacterial culture, or evidence of extra-abdominal infection at the time of ascitic fluid collection for this study. Prior to fluid collection 8 patients had previously had SBP, including the 2 diagnosed in the 2 days before, but only 2 had organisms cultured using conventional microbiological culture techniques (*E*. *coli* and *Streptococcus mitis*). The median length of time between patients’ most recent episode of SBP and the paracentesis for this study was 86 days (IQR 22.3–174.3). Sixteen patients (64%) had received antibiotics within 2 weeks of the paracentesis: SBP prophylaxis (n = 7); intravenous broad spectrum antibiotics <48 hours prior to sample collection (n = 7) or ≤7 days prior to sample collection (n = 2); other oral antibiotic courses (n = 3, 1 for *Helicobacter pylori* eradication (7 days), 1 for cellulitis prophylaxis (120 days) and 1 was patient directed, taking antibiotics 1 day before paracentesis). There was no significant difference in 16S levels between patients who had antibiotics during the previous 2 weeks and those who had not (p = 0.28, Fig. 2A).

Bacterial burden was weakly correlated with the number of neutrophils in the ascitic fluid (r_s_ = 0.5, p = 0.012) (Fig. 2B), but did not correlate with serum markers of infection/inflammation (C reactive protein (p = 0.44) or procalcitonin (p = 0.52)). Patients with a prior diagnosis of SBP had significantly higher CFU/ml (p = 0.027, Fig. 2C), particularly those taking oral prophylaxis. Bacterial burden was negatively correlated with ascites total protein level (r_s_ = -0.42, p = 0.045, Fig. 2D), but not with serum markers of renal impairment (urea, creatinine, sodium) or liver failure (bilirubin or CTP score).

As highlighted in Table 1 and Fig. 1, clinical outcomes for patients with ascites were poor. Bacterial DNA burden was associated with a shorter time to readmission (r_s_ = -0.50, p = 0.024, Fig. 2E), and was significantly higher in patients who died or developed SBP within 6 months (p = 0.006, Fig. 2F). Although 2 patients had a diagnosis of SBP 2 days prior to inclusion into the study, they were culture negative and non-neutrocytic at the time of sampling (19 and 33 PMN/ml) and there was nothing in their clinical features or history to distinguish them (6 other patients had previous SBP, 7 were treated with IV antibiotics around the time of sampling). Analysing the data after excluding these patients produced the same results reported here.

The most significant clinical parameters associated with death or subsequent development of SBP were mean arterial blood pressure (MAP, p = 0.007) and low levels of ascites total protein (p = 0.021), which was also associated with high bacterial burden (Fig. 2D). There was a trend towards significantly worse outcomes for patients who had a previous episode of SBP (p = 0.056), but there were no differences for markers of renal or liver failure mentioned above. Prescription of propranolol, lactulose, proton pump inhibitors (PPI) or diuretics did not differ between the 2 cohorts (all p>0.3).

### Ascites bacterial community composition

**Fig 3:**
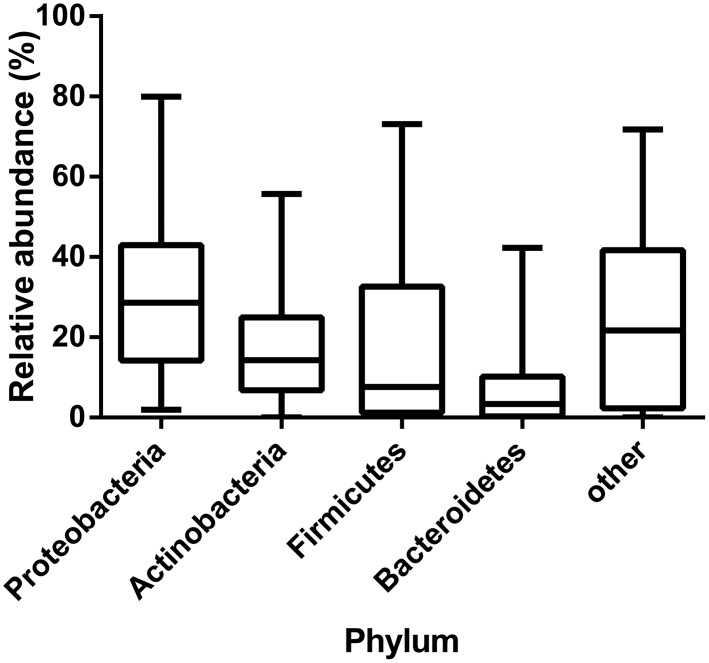
Distribution of bacterial phyla identified by 16S sequence analysis of ascites bacterial DNA. Boxes represent median values and error bars show interquartile range

16S rRNA gene amplicon sequences were used to assess bacterial community composition. Bacterial phyla comprised both Gram positive and Gram negative taxa. In keeping with previous studies, the most commonly detected phylum was Proteobacteria (median relative abundance 28.6%), followed by Actinobacteria (14.3%), Firmicutes (7.7%), and Bacteroidetes (3.4%), with no other single phylum representing more than a median value of 0.39% of total abundance (Fig. 3). When assessed at the genus level, samples had a median taxon richness of 23 (range: 9–96, IQR15-37.5). The most commonly detected genera included *Streptococcus* (15/25 patients), *Porphyromonas* (12/25 patients), and *Enterobacter* (12/25 patients). A significant correlation between relative genus abundance and frequency of detection (the number of patients a genus was detected in) was observed (Spearman’s r = 0.59, p<0.0001). Bacterial community composition differed substantially between patients, with a genus level mean BC score of 0.06 ± 0.06 (where a score of 1 indicates identical communities, and 0 indicates no similarity). Further, no significant clustering was identified by NMS, suggesting a stochastic community assembly. Neither were statistically significant correlations identified between bacterial community composition, or community diversity measures and categorical or continuous clinical variables.

### Ascites fluid leukocyte composition and phenotype

**Fig 4:**
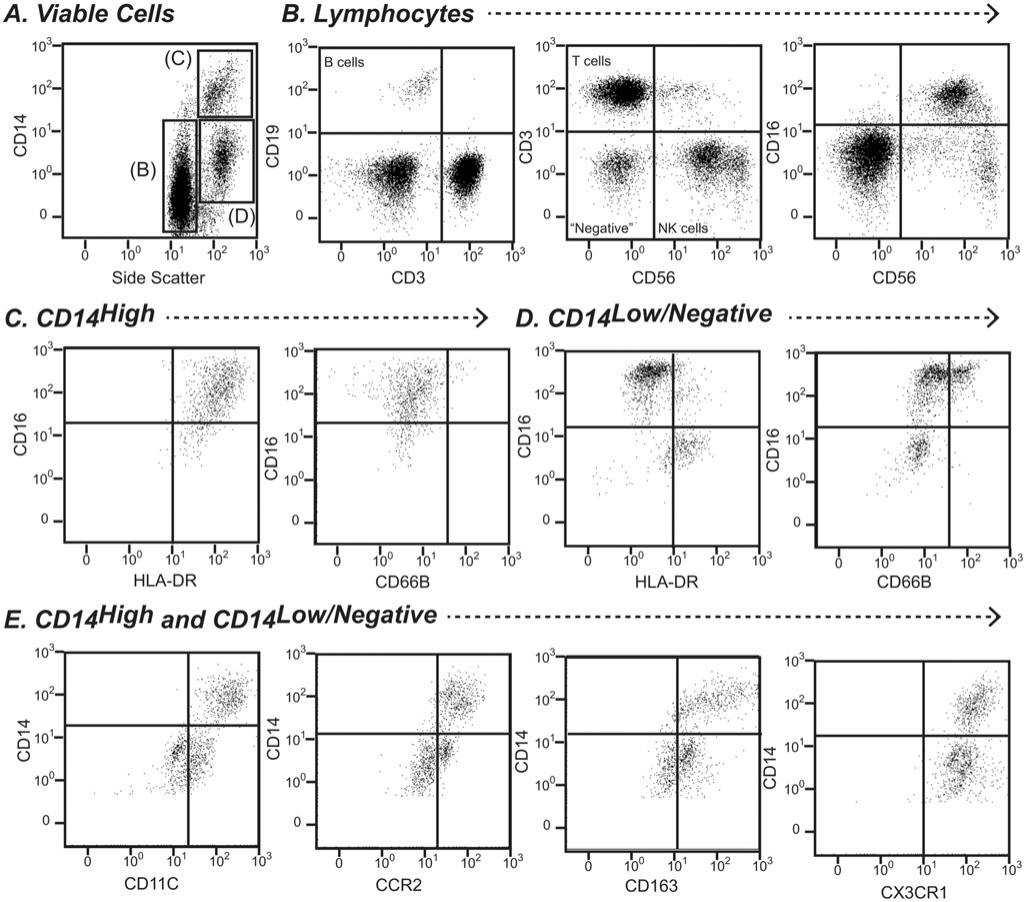
Flow cytometry gating strategy for ascites leukocyte characterisation. (A) Lymphocytes and myeloid cells were distinguished on the basis of side scatter properties and CD14 expression. CD14Hi and CD14 Low/^negative^ cells were further characterised for CD16, HLA-DR and CD66B expression (top right panels). Lymphoid cells were classified as B cells (CD19+), T cells (CD3+), and NK cells (CD56+/CD16+/-) (bottom left panels). (B) Myeloid populations were further investigated for surface CD11C, CCR2, CD163 and CX3CR1 expression.

**Fig 5:**
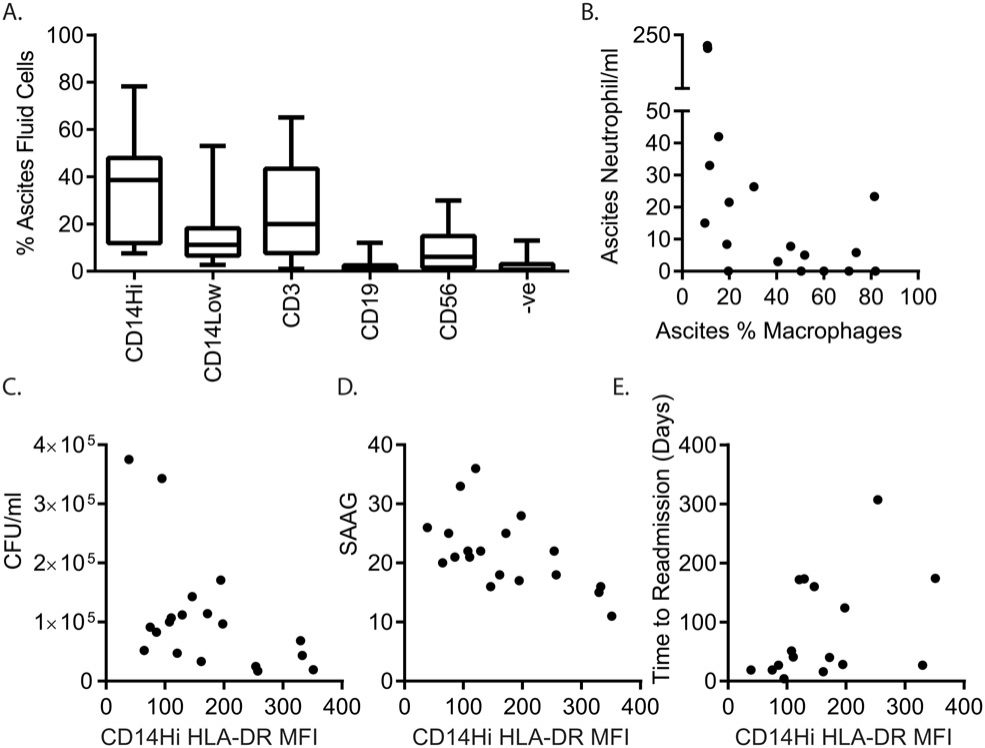
Ascites bacterial DNA burden associated with reduced HLA-DR expression on ascitic fluid macrophages. (A) distribution of leukocyte lineages in ascites fluid (n = 18, midline represents median, box represents 25^th^-75^th^ percentile, whiskers indicate minimum and maximum values,-ve indicates lack of staining for markers employed in this study) (B) Correlation between %CD14+ macrophages and neutrophil count in ascitic fluid. Correlation between surface HLA-DR expression on CD14+ ascites cells (MFI) and (C) ascites bacterial burden (CFU/ml), (D) serum ascites albumin gradient (SAAG) and (E) time to next hospital admission.

Ascites immune cells from 18 patients were profiled using a panel of markers to enumerate T cells (CD3+), B cells (CD19+), monocytes/macrophages (CD14+), and natural killer (NK) cells (CD56+), as well as staining for CD16, HLA-DR and the granulocyte activation marker CD66B to further phenotype cells of interest (Fig. 4). These lineage markers typically accounted for >95% of ascites cells. The most abundant leukocytes in ascites fluid were generally CD14^High^ macrophages (median 38.6%), with lower numbers of SSC^High^/CD14^Low/-ve^ myeloid cells (median 11.2%), T cells (median 19.9%), NK cells (median 6.1%), and B cells (median 1.1%) (Fig. 5A). Unlike peripheral blood monocytes, a minority of which express CD16 (approximately 10%), the majority of ascites CD14^High^ cells co-expressed CD16; and essentially all expressed HLA-DR. CD16^-^ and CD16^+^ macrophage populations were not clearly discernible (Fig. 4C), however there was a broad spectrum of CD16 expression. SSC^High^/CD14^Low/neg.^ cells were subdivided on the basis of HLA-DR expression. CD14^Low/neg.^/HLADR^neg.^ cells were typically CD16^+^, with a variable proportion of CD66B^+^ cells (activated granulocytes). The CD14^Low/neg.^/HLA-DR^+^ population may contain monocytes/macrophages with low HLA-DR expression; CD16 expression in the population varied, but all were CD66B^Neg.^ (Fig. 4D). We further phenotyped ascites myeloid cells from 7 patients, using a panel of markers that distinguish blood monocyte subsets. Greater than 90% of ascites CD14^High^ macrophages co-expressed CCR2, CX3CR1, CD163 and CD11c. CD14^Low/-neg.^ cells were CX3CR1+, and a proportion expressed CCR2, and CD163 and CD11c at low levels, consistent with the presence of a CD14^Low/neg.^ monocyte/macrophage population (Fig. 4E).

The ascites lymphocyte compartment was not characterised in detail in this study, however we did observe a high proportion of T cells expressing the NK cell-associated receptor CD56+ in a subset of patients (Median 20%, (range 1–50%), which can be induced by T cell receptor stimulation, and has been shown to mark a subset of T cells with major histocompatibility complex-unrestricted cytotoxicity[14] (Fig. 4B). Ascites NK cells could be subdivided into CD56^High^/CD16^-^, immunoregulatory cells, and CD56^Low^/CD16^+^, cytotoxic, cells, however their relative proportions were extremely variable (CD56^High^/CD16^-^ Median 8% (range 0–47%)) (Fig. 4B). CD56^Low^/CD16^+^ cells comprise the majority of peripheral blood NK cells (90%), whereas CD56^High^/CD16^-^ NK cells, thought to be the direct precursors of CD56^Low^/CD16^+^ cells, dominate secondary lymphoid organs and tissues[15]. With the exception of NK cells, leukocyte frequency in ascites fluid was not related to their proportions in peripheral blood collected at the time of paracentesis (data not shown).

### High ascites bacterial burden is associated with reduced macrophage HLA-DR expression

The presence of monocytes/macrophages (CD14^+^/HLA-DR^+^) in ascites fluid was associated with lower ascites neutrophil numbers and a trend towards lower bacterial burden (r_s_ = -0.59, p = 0.011 and r_s_ = -0.41, p = 0.081 Fig. 5B and data not shown). Consistent with the hypothesis that innate immune function is impaired in patients with cirrhosis, HLA-DR expression on ascites CD14^Hi^/HLA-DR^+^ monocytes/macrophages inversely correlated with bacterial DNA levels (r_s_ = -0.48, p = 0.04, Fig. 5C). Similar to the association between high bacterial DNA levels and low ascites total protein content, macrophage HLA-DR expression inversely correlated with the serum ascites albumin gradient (SAAG, rs = -0.59, p = 0.01 Fig. 5D), indicative of low ascites total protein and portal hypertension. Like bacterial DNA burden, low macrophage HLA-DR expression was associated with shorter time to readmission (rs = 0.546, p = 0.036, Fig. 5E). There was a non-significant trend towards lower macrophage HLA-DR expression in patients who had previous episodes of SBP, and in those who died during the 6 months follow-up. Interestingly, patients’ ascitic macrophage HLA-DR expression was not related to HLA-DR expression on their peripheral blood monocytes. Although minor in proportion, B lymphocytes were the only cell type whose frequency was positively correlated with ascites bacterial burden (r_s_ = 0.65, p = 0.003), however B cell HLA-DR expression did not correlate with bacterial burden.

## Discussion

Bacterial infection is a major cause of early death in patients with cirrhosis. Previous studies have reported the presence of bacterial DNA in ascites fluid, even in culture-negative and non-neutrocytic ascites[4,16], and confirmed at least some of this DNA is associated with viable bacteria[17]. However, the clinical significance of ascites bacterial DNA has not been widely studied. We report both the detection of bacterial DNA in a high proportion of culture negative, non-neutrocytic ascites fluid (23/25 patients) and a positive association between levels of bacterial DNA and poor clinical outcomes, including readmission, and death. Whether the presence of bacterial DNA in ascites fluid represents a sub-clinical or pre-clinical infection, or simply reflects the severity of other features of liver disease, such as portal hypertension or intestinal imbalance, is not clear. A previous report that ascites bacterial DNA was associated with short term mortality, but not with infection, would support the latter interpretation[18]. Moreover, portal hypertension has been suggested as a cause of intestinal permeability and bacterial translocation, and plasma levels of bacterial DNA were associated with systemic circulatory abnormalities in cirrhotic patients with ascites[19].

In keeping with previous studies, the ascites bacterial composition reported here comprises a broad phylogenetic range, including Gram positive and Gram negative species, with a predominance of Proteobacteria and a high relative abundance of Actinobacteria, Firmicutes, and Bacteroidetes. These phyla represent the four most commonly associated with the human microbiota, and those which typically dominate commensal communities in the gut and elsewhere. The relative abundance of the phyla detected in ascitic fluid differs from that seen in the gut of healthy individuals, where Bacteroidetes and Firmicutes typically dominate[20]. This distribution suggests that translocation of bacteria to the peritoneal cavity is not limited to those present in the gut, or that the gut microbiota in this patient population differs from that in healthy individuals. There is evidence to support each of these models. Qin and co-workers recently described intestinal dysbiosis in cirrhotic patients, and reported that the majority of patient-enriched species were of buccal origin, suggesting translocation from the mouth to the gut occurs in cirrhosis[21]. It has also been shown that in patients with cirrhosis, there is shift in the composition of the gut microbiota, with a decrease in the relative abundance of Bacteroidetes and an increase in the relative abundance of Proteobacteria and Fusobacteria[22]. Analysis of detected bacteria at the genus level revealed the genera *Streptococcus*, *Porphyromonas*, and *Enterobacter* were commonly present, in keeping with translocation from the oral cavity or intestine. The absence of significant correlations between the bacterial composition of the samples analysed and clinical markers of disease is notable. NMS analysis indicated that there was low similarity between the profiles generated from individual patients. The absence of a consensus microbiota composition is likely to reflect the heterogeneous patient population, the multifactorial nature of cirrhosis, and the stochastic nature of bacterial translocation from areas of highly complex bacterial microbiota. Given this high degree of inter-individual heterogeneity, longitudinal studies in larger cohorts, with parallel sequencing of intestinal microbiota, will be required to investigate the relationship between ascites bacterial composition, immunity and clinical outcomes, including infection.

The common detection of low level bacterial DNA in ascites, and the previous reports of the detection of viable bacterial cells using culture-independent methodologies in patients without peritonitis, suggests that translocation of bacteria into the peritoneal cavity is necessary, but not sufficient, to trigger SBP. Impaired innate immune function in cirrhotic patients is likely to contribute to susceptibility to infection. Previous studies have reported low levels of HLA-DR expression on peripheral blood monocytes in critically ill patients with cirrhosis[23,24] and acute liver failure[25]. We observed reduced HLA-DR expression on ascites (but not peripheral blood) macrophages/monocytes, which was associated with increased ascites bacterial DNA burden. The presence of macrophages per se, however, correlated with reduced ascites neutrophils and bacterial DNA, suggesting that restoring macrophage function (e.g. HLA-DR expression) may be a viable therapeutic strategy. Diminished monocyte HLA-DR expression, which compromises T cell activation[26], is a well-established biomarker of a transient, compensatory anti-inflammatory response syndrome that occurs in response to infection or systemic inflammation[27]. This post-inflammatory immunodeficiency, in which innate immune cells become refractory to further stimulation, prevents inflammation-induced injury, but can also predispose patients to lethal infection. The phenomenon is also known as endotoxin tolerance, although it is not specific to endotoxin, as other exogenous and endogenous stimuli can induce tolerance. The mediators and mechanisms of endotoxin tolerance are not fully understood, and different mechanisms underlie different aspects of the phenotype[28,29]. IFNγ and GM-CSF[29–31] have been reported to restore HLA-DR expression and phagocytic function in vitro and in vivo, including in patients with cirrhosis[30], however host inactivation of the inflammatory stimulus, in particular endotoxin, can also be required for recovery from the tolerant state[32]. The mechanisms underlying reduced HLA-DR expression in ascites, the duration of the phenotype and the functional implications warrant further investigation.

Clinical evidence supports the use of antibiotics for SBP treatment and prophylaxis, but how antibiotics impact on the gut and ascites microbiota is yet to be defined. Changing bacterial and resistance patterns in patients with cirrhosis have been attributed to the increasing use of antiobiotic prophylaxis and invasive procedures, highlighting the importance of ensuring appropriate antibiotic use[33] Long term use of broad-spectrum antibiotics may lead to an increase in pathogenic bacteria in the gut and increased antibiotic resistance[33–35]. Whether adjunct approaches such as pro- or pre-biotic treatment to restore gut homeostasis, or immunomodulatory approaches to boost immunity, without exacerbating immunopathology, could be useful in this cohort remains to be evaluated. Approaches that reduce the burden of endotoxin (or other inflammatory stimuli), or restore HLA-DR expression and immune function (e.g. IFNγ, GM-CSFor albumin treatment [30,36,37]) may be viable therapeutic strategies. Immune monitoring protocols are also needed to identify patients who may benefit from such interventions.

In conclusion, we report that the presence of ascites bacterial DNA in patients with decompensated cirrhosis and ascites is associated with early death and readmission, and is an indicator of impaired immunity, which may contribute to susceptibility to infection in these patients. Whether the presence of DNA, or other bacterial products, is indicative of a sub-clinical infection, or simply a persistent source of inflammatory stimuli that exacerbates liver pathology, remains to be clarified. Characterisation of ascites bacteria, their source and role in infection and inflammation, as well as the contribution of host immune factors, is crucial to developing effective treatment regimens, and minimising antibiotic resistance.
